# Quercetin Attenuates Osteoporosis in Orchiectomy Mice by Regulating Glucose and Lipid Metabolism *via* the GPRC6A/AMPK/mTOR Signaling Pathway

**DOI:** 10.3389/fendo.2022.849544

**Published:** 2022-04-25

**Authors:** Jie Sun, Yalan Pan, Xiaofeng Li, Lining Wang, Mengmin Liu, Pengcheng Tu, Chengjie Wu, Jirimutu Xiao, Qiuge Han, Weiwei Da, Yong Ma, Yang Guo

**Affiliations:** ^1^Laboratory of New Techniques of Restoration & Reconstruction of Orthopedics and Traumatology, Nanjing University of Chinese Medicine, Nanjing, China; ^2^School of Nursing, Nanjing University of Chinese Medicine, Nanjing, China; ^3^Department of Orthopedics and Traumatology, Shanghai Municipal Hospital of Traditional Chinese Medicine, Shanghai University of Traditional Chinese Medicine, Shanghai, China; ^4^School of Chinese Medicine, School of Integrated Chinese and Western Medicine, Nanjing University of Chinese Medicine, Nanjing, China; ^5^ Mongolian Medicine College, Inner Mongolia Medical University, Hohhot, China

**Keywords:** quercetin, glucose metabolism, lipid metabolism, androgen deprivation therapy-induced osteoporosis, Gprc6a/AMPK/mTOR signaling pathway

## Abstract

Quercetin, a flavonoid found in natural medicines, has shown a role in disease prevention and health promotion. Moreover, because of its recently identified contribution in regulating bone homeostasis, quercetin may be considered a promising agent for improving bone health.** **This study aimed to elucidate the role of quercetin in androgen deprivation therapy-induced osteoporosis in mice. C57BL/6 mice were subjected to orchiectomy, followed by quercetin treatment (75 and 150 mg/kg/d) for 8 weeks. Bone microstructure was then assessed by micro-computed tomography, and a three-point bending test was used to evaluate the biomechanical parameters. Hematoxylin and eosin (H&E) staining was used to examine the shape of the distal femur, gastrocnemius muscle, and liver. The balance motion ability in mice was evaluated by gait analysis, and changes in the gastrocnemius muscle were observed *via* Oil red O and Masson’s staining. ELISA and biochemical analyses were used to assess markers of the bone, glucose, and lipid metabolism. Western blotting analyses of glucose and lipid metabolism-related protein expression was performed, and expression of the GPCR6A/AMPK/mTOR signaling pathway-related proteins was also assessed. After 8 weeks of quercetin intervention, quercetin-treated mice showed increased bone mass, bone strength, and improved bone microstructure. Additionally, gait analysis, including stride length and frequency, were significantly increased, whereas a reduction of the stride length and gait symmetry was observed. H&E staining of the gastrocnemius muscle showed that the cross-sectional area of the myofibers had increased significantly, suggesting that quercetin improves balance, motion ability, and muscle mass. Bone metabolism improvement was defined by a reduction of serum levels of insulin, triglycerides, total cholesterol, and low-density lipoprotein, whereas levels of insulin-like growth factor-1 and high-density lipoprotein were increased after quercetin treatment. Expression of proteins involved in glucose uptake was increased, whereas that of proteins involved in lipid production was decreased. Moreover, the GPRC6A and the phospho-AMPK/AMPK expression ratio was elevated in the liver and tibia tissues. In contrast, the phospho-mTOR/mTOR ratio was reduced in the quercetin group. Our findings indicate that quercetin can reduce the osteoporosis induced by testosterone deficiency, and its beneficial effects might be associated with the regulation of glucose metabolism and inhibition of lipid metabolism *via* the GPCR6A/AMPK/mTOR signaling pathway.

## Introduction

Androgens, including testosterone and its precursors, are vital for the musculoskeletal and endocrine-reproductive systems (1). In men 30–90 years of age, the testosterone levels slowly decrease by 1% annually (2, 3). Similarly, chronic illnesses, such as diabetes and obesity, and endocrine treatments, such as androgen deprivation therapy (ADT) after prostate cancer, can alter testosterone levels (1, 4). Low testosterone levels, referred to as “testosterone deficiency”, can result in several symptoms, including erectile dysfunction, lack of energy or tiredness, depression and anxiety, bone mineral density (BMD) decline, and hair loss (1, 5, 6). These symptoms impair the lives of older men; thus, testosterone deficiency is considered a serious public health concern in the current aging society.

Similar to estrogen deficiency, the apparent relationship between bioavailable serum testosterone levels and the etiology of osteoporosis in elderly men has been clarified. A recent study examining 5540 subjects 20–59 years of age found that the lumbar BMD was positively associated with serum testosterone levels (7). Moreover, after excluding the main risk factors for fractures, serum testosterone levels were also correlated closely with fracture risk (8). The negative impact of testosterone reduction on the bone health is more unequivocal in the setting of ADT. A recent study in Korea showed that patients receiving ADT were more inclined to have newly developed osteoporosis and osteoporotic fractures (4). Thus, testosterone performs a central role in maintaining BMD and musculoskeletal health in older men.

Although the therapeutic regimens of testosterone deficiency-induced osteoporosis has not yet been fully established, the effectiveness of testosterone combined with bisphosphonate is well established (9). However, despite its beneficial effects on bone health, testosterone therapy may cause other problems, including decreased sperm production and fertility, cardiovascular disease, and prostate cancer or hyperplasia (1, 9).

Owing to the increased risk of disease from testosterone replacement therapy, investigators have shifted their focus to natural compounds that promote testosterone secretion and bone quality. Quercetin, a natural flavonol found in herbal medicines, as well as in fruits and vegetables (10). Quercetin possesses various pharmacological benefits, including antioxidative, anti-inflammatory, and antiapoptotic effects (10, 11). Recent studies have also shown that quercetin can promote testosterone secretion and ameliorate ovariectomy-induced osteoporosis *in vitro* and *in vivo* (12–15). Furthermore, it can decrease blood pressure, hyperlipidemia, and hyperglycemia, thus addressing the key problematics of testosterone therapy. However, the literature on quercetin and testosterone deficiency-induced osteoporosis is very limited.

The G-protein-coupled receptor class C group 6 member A (GPRC6A), expressed in the liver and muscles, is closely involved in the regulation of glucose and lipid metabolism (16–18). Previous research has found that GPRC6A(-/-) male mice exhibit osteoporosis and metabolic syndrome, such as increased weight and higher fat content, hyperglycemia, hyperphosphatemia, hypercalciuria, and feminization (17). In addition, computer simulations support that quercetin binds to GPCR6A and may trigger related genomic-mediated effects (19). Thus, GPRC6A may provide a novel insight into the molecular basis of quercetin treatment for testosterone deficiency-induced osteoporosis.

Here, orchiectomy was used to generate a testosterone deficiency mouse model and verify the potential therapeutic role of quercetin on testosterone deficiency-induced osteoporosis. To elucidate the possible mechanisms underlying osteoporosis induced by testosterone deficiency and the bone-protective effects of quercetin, relevant markers of glucose and lipid metabolism and components of the GPCR6A/AMPK/mTOR signaling pathway were investigated.

## Materials and Methods

### Animals

In the standard barrier facility of the Experimental Animal Center of Nanjing University of Chinese Medicine, 48 male C57BL/6 mice (12 weeks old) were fed and raised. The animal room had a controlled temperature: 24 ± 2°C, and humidity: 60 ± 2%, with a 12:12 h dark-light cycle. Animal studies described in this manuscript have been approved by the Nanjing University of Chinese Medicine Institutional Animal Care and Use Committee (NO. ACU170804).

### Orchiectomy Procedure and Experimental Grouping

Orchiectomy is a typical model of osteoporosis secondary to testosterone deficiency. As in a previous study (20), the surgical procedure was performed under sterile conditions, and all mice were anesthetized by an intraperitoneal injection of 1% sodium pentobarbital (60 mg/kg body weight). A ventral midline incision of approximately 1.5 cm was made in the scrotum to expose the tunica after skin preparation. The connective tissue was gently teased away, the blood vessels supplying the testes were isolated and ligated, and the testis and epididymis were removed through the incision. Finally, tetracycline ointment was applied to prevent infection after the incision was closed. In the sham-operated group, surgical procedure similar to that used for the removal of the testis and epididymis was utilized.

All treatment dosing regimens began 8 weeks after surgery. The sham-operated group (sham, n=12) received saline at a dose of 0.001 mL/kg/d. In addition to the sham-operated group, mice that underwent orchiectomy (n=36) were randomly divided into four groups. For this study, we set two different concentrations and the mice were grouped as follows: (a) the model group (model) and the sham-operated group (sham) received equivalent volumes of saline, (b) the low-quercetin group (QL) received 75 mg/kg/d of quercetin, and (c) the high-quercetin group (QH) received 150 mg/kg/d of quercetin. Saline or drugs were administered by gavage once daily for 8 weeks. The mice were anesthetized for blood collection from the retro-orbital vein and then euthanized. The liver, gastrocnemius, femur, and tibia were removed and collected carefully. The right gastrocnemius, femurs, tibias were stored in 4% paraformaldehyde, while the left gastrocnemius, femurs, tibias were stored in -80°C for further measurement.

### Gait Analysis

Gait analysis was conducted using a ventral plane videography instrumentation (MSI, USA). All mice first ran at least seven adaptive runs as training before being formally recorded. After the adaptive runs, the mice were exercised and recorded by the camera at a running speed of 12 cm/s in the same direction. Stride length, stride length coefficient of variation (CV), stride frequency, and gait symmetry were analyzed to assess the ability of dynamic balance.

### Micro-Computed Tomography (Micro-CT)

For bone microarchitecture analysis of the trabecular bone, micro-CT (Skyscan 1176, Germany) was used. The parameters were set as follows: voxel size 9 μm, voltage 55 kV, and current 70 mA. After completion of the scan, the region of interest (ROI) was selected from a distance of 0.3 to 0.6 mm from the highest point on the distal femur growth plate. The 3D images were analyzed and the bone features, including BMD, volume/tissue volume (BV/TV), trabecular thickness (Tb.Th), trabecular number (Tb.N), and trabecular separation (Tb.Sp), were measured.

### Biomechanical Parameter Analysis

The right femurs was collected for the biomechanical three-point bending test and performed on a servohydraulic test system (MTS acumen3, USA) at 0.01 mm/s with peak load and recorded. The biomechanical parameter of stiffness, maximum load, maximum deflection, and fracture energy were then calculated from displacement and force.

### Hematoxylin and Eosin (H&E) Staining

The femur was decalcified in a decalcifying liquid for 4–6 weeks after fixation in 4% paraformaldehyde, whereas the liver and gastrocnemius muscle were only fixed in 4% paraformaldehyde prior to the next step. Next, the femur, liver, and muscle were dehydrated by gradient ethanol, and then cleared with xylene solution. Specimens were then embedded in paraffin, and serial sections were generated at 5 µm thickness. The sections were stained with hematoxylin and eosin. Then sections observe alterations in the bone microstructure through an inverted microscope (Leica, DM1000).

### TRAP Staining

TRAP staining kit (Sigma, 387A)was used and the specific experimental steps were performed as the manufacturer’s instructions and as previously described (21). The osteoclast area to bone surface (OCs/BS) ratios were quantified.

### Masson’s and Oil Red O Staining

The sections were prepared as before and then stained according to the protocol of Masson’s staining kit protocol (Yeasen, 60532ES58). The red stain represented the muscle fibers and blue stains represented the collagen fibers, and the muscle fiber area and collagen fiber area were calculated.

The gastrocnemius muscle and liver were fixed and embedded in the OCT compound. Next, the slices were placed in Oil Red O solution for 8 min for staining. The slices were washed twice with 60% isopropyl alcohol and re-stained by hematoxylin. Finally, the slices were photographed, and the area of the lipid droplet formation was analyzed.

### ELISA

All kits were provided by Nanjing Jin Yibai Biological Technology Company (China). The expression serum of testosterone (Jin Yibai, JEB-12629), β-isomer of C-terminal telopeptide of type I collagen (β-CTX, Jin Yibai, JEB-12299), TRAP (Jin Yibai, JEB-12833), IL-6 (Jin Yibai, LA128802H), bone alkaline phosphatase (B-ALP, Jin Yibai, JEB-12359), Osteocalcin (OCN, Jin Yibai, JEB-17685), insulin-like growth factor-1 (IGF-1, Jin Yibai, JEB-12259), and insulin (Mercodia, 10-1247-01) were measured based on the standard sandwich ELISA protocol.

### Biochemical Analysis

Triglyceride, total cholesterol, high-density lipoprotein (HDL), low-density lipoprotein (LDL) and free fatty acid (FFA) levels were detected using the automatic biochemical analyzer (AU680, Beckman).

### Western Blotting

The left tibia, gastrocnemius muscle and liver was lysed in RIPA lysis buffer containing a protease inhibitor cocktail. The samples were sonicated and incubated on ice for 30 min and then the insoluble material was removed by centrifuging at 12000 rpm for 5min. The protein in the supernatant was collected and then quantified by BCA kit (Yeasen, 20201ES76). A 10% SDS-PAGE was used, and electrophoresis was performed with a voltage of 140 V. Subsequently, the gel was transferred onto PVDF membranes with a constant electric current of 400 mA. After the PVDF membranes were blocked using blocking solution for 15 min, the membranes were incubated with anti-Gprc6a (1:4000; SAB, 47990), anti-AMPK (1:4000; CST, 2757), anti-p-AMPK (1:2000; CST, 50081), anti-RUXN2 (1:4000; Proteintech, 20700-1-AP), anti-Osterix (1:4000; Abcam, ab94744), and anti-GAPDH (1:5000; Proteintech, 10494-1-AP) overnight at 4°C and then incubated with the secondary antibody (1:10000; Proteintech, 20536-1-AP) for 2 h on a horizontal shaker at normothermia. Finally, the membrane was moistened using ECL reagent and the image was exposed to obtain the bands.

### Quantitative Real-Time PCR (qRT-PCR)

Total RNA was extracted using the Bone RNA Kit and Cell/Tissue Total RNA Kit (Yesaen, 19211ES60), and then 1st Strand cDNA Synthesis Kit (Yesaen, 11119ES60) was used to RNA reverse transcription. The Hieff qPCR SYBR Green PCR Master Mix (Yesaen, 11201ES03) was used for q-PCR analysis. Each value was adjusted using β-actin as the reference. The 2^- ΔΔCt^ method was used to analyze PCR array results. The qPCR primers sequences are as follows:OCN: Forward 5’ CTGAAAAGCCCACAGATACCAG3’ and Reverse 5’ TGGAGAGGGTTGTTAGTGTGTC3’. RUNX2: Forward 5’ ATGCTTATTCGCCTCACAAA3’ and Reverse 5’ GCACTCACTGACTCGGTTGG3’. Osterix: Forward 5’ ATGGCGTCCTCTCTGCTTG3’ and Reverse 5’ TGAAAGGTCAGCGTATGGCTT3’ β-actin: Forward 5’ GGCTGTATTCCCCTCCATCG 3’ and Reverse 5’ CCAGTTGGTAACAATGCCATGT 3’.

### Statistical Analysis

The raw data and generated statistics were analyzed using the SPSS software (v 23.0). The data were transformed and presented as the mean ± standard deviation (SD) and were analyzed using the Kruskal–Wallis one-way analysis of variance (ANOVA) when the data were not normally distributed. Differences were considered significant when P < 0.05. The GraphPad Prism software 9.0.0 was used to generate the figures.

## Results

### Changes in the Serum Levels of Testosterone

**Figure 1:**
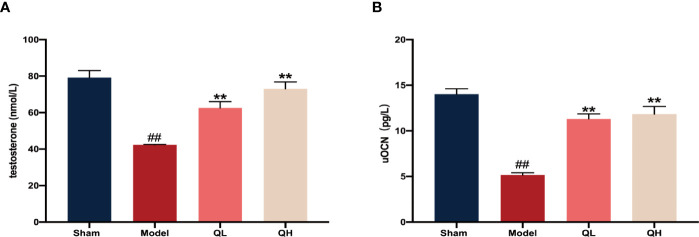
Changes in serum levels of testosterone and undercarboxylated osteocalcin (uOCN) in mice that have undergone orchiectomy. **(A)** Serum testosterone levels in the sham-operated (sham), model (model), low- (QL), and high-quercetin groups (QH). **(B)** Serum uOCN levels in all treatment and control groups. ##*p<*0.01 vs. sham group; **p<0.01 vs. model group.

Upon orchiectomy, due to the absence of the testes and epididymides, the serum testosterone levels decreased rapidly. As expected, the testosterone levels increased after 8 weeks of quercetin treatment both for the low-quercetin group (QL, 75 mg/kg/d) and the high-quercetin group (QH, 150 mg/kg/d) as compared with the model group receiving saline solution (*p<*0.01, **Figure 1A**). Simultaneously, the serum levels of undercarboxylated osteocalcin (uOCN), which promotes testosterone secretion, increased accordingly (*p<*0.01, **Figure 1B**).

### Quercetin Improves Bone Microstructure Upon Orchiectomy

**Figure 2:**
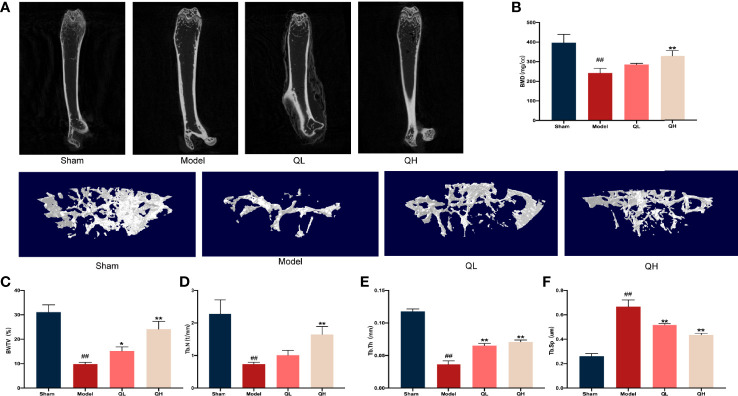
Quercetin induced improvement of bone microstructure in mice that underwent orchiectomy. **(A)** 2D images and 3D reconstruction of the distal femur by micro-computed tomography. **(B)** Bone mineral density **(**BMD) of the distal femur. **(C–F)** Changes in bone characteristics, including bone volume/tissue volume (BV/TV), trabecular number (Tb. N), trabecular thickness (Tb. Th), and trabecular separation (Tb.Sp). ##*p<*0.01 vs. sham group; *p<0.05, **p<0.01 vs. model group.

**Figure 3:**
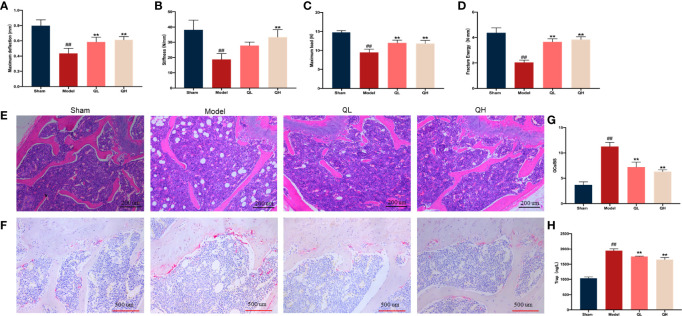
Effects of quercetin on the biomechanical characteristics and capacity for bone resorption in mice that underwent orchiectomy. **(A–D)** Changes in the biomechanical characteristics in femur: including the maximum load and deflection, fracture energy, and stiffness. **(E)** The effects of quercetin on bone microstructure in the distal femoral bone, visualized by H&E staining. **(F)** Effects of quercetin on the bone resorption in mice that underwent orchiectomy, visualized by TRAP staining. **(G)** Osteoclast surface/bone surface (OC.s/BS). **(H)** Changes in serum levels of TRAP in mice that have undergone orchiectomy. ##*p<*0.01 vs. sham group; **p<0.01 vs. model group.

To measure the bone volume and bone microstructure in mice that underwent orchiectomy, micro-CT scans were employed and the characteristics of the distal femoral trabecular bone were evaluated. As shown in **Figure 2A**, the bone trabeculae was significantly lower in the model group than the sham-operated group. Further data analysis showed that volume/tissue volume (BV/TV) and trabecular thickness (Tb.Th) were higher and trabecular separation (Tb.Sp) was markedly lower in both the low- and the high-quercetin groups than in the model group (*p*<0.01); additionally, the high-quercetin group had significantly increased bone mineral density (BMD, *p*<0.01) and trabecular number (Tb.N, *p*<0.01) levels (**Figures 2B** **–F**). Next, we investigated the microstructure of the distal femoral trabecular bone using H&E staining (**Figure 3E**). The distal femoral had lesser bone trabecula and thinner cortical bones in the model group than the sham-operated group. In addition, increased bone trabecula, fewer lipid droplets, and better intertrabecular connectivity were observed in the low- and high-quercetin groups. These data suggest that after 8 weeks of treatment, bone density improved and the high-dose quercetin effectively increased BMD and improved bone microarchitecture in mice that had undergone orchiectomy. In addition, bone resorption of the distal femoral trabecular bone was observed directly by TRAP staining (**Figures 3F**). We found that quercetin administration alleviated bone loss after orchiectomy in mice and decreased the OCs/BS ratio (**Figures 3G**). Subsequently, the levels of serum TRAP were measured and the changes after quercetin administration is consistent with TRAP staining (**Figures 3H**).

### Quercetin Improves Biomechanical Parameters Upon Orchiectomy

Fresh femurs were evaluated using a three-point bending test to determine the biomechanical status of the cortical bone. The maximum load and deflection, fracture energy, and stiffness were significantly lower in the model group than in the sham-operated group (**Figures 3A** **–D**) and increased in both the low- and high-quercetin groups when compared with the model (*p*<0.01). In addition, stiffness was significantly higher in the high-quercetin group than in the model group (*p*<0.01).

### Quercetin Improves the Balance Motion Ability Upon Orchiectomy

**Figure 4:**
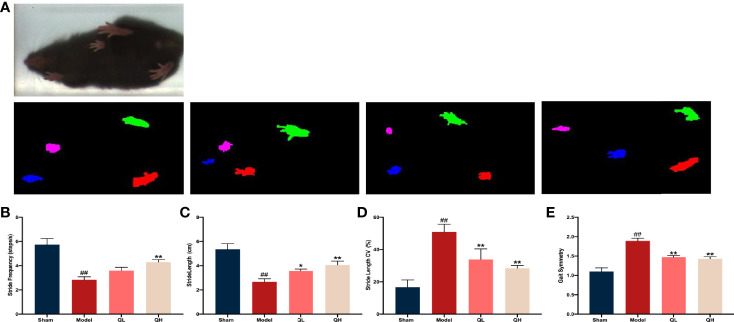
Gait analysis in mice that underwent orchiectomy. **(A)** The full gait was recorded and segmented into independent gait cycles. Changes in gait characteristics: including the **(B)** stride length, **(C)** stride length CV, **(D)** stride frequency, and **(E)** gait symmetry. ##*p<*0.01 vs. sham group; *p<0.05, **p<0.01 vs. model group.

The musculoskeletal system is a dynamic system that requires a strong balance. A decreased compliance of the body was observed in the muscle system when the skeletal system was changed. After treatment with high dose of quercetin, we found that the stride length and frequency significantly increased (*p*<0.01), whereas stride length CVs and gait symmetry decreased correspondingly (*p*<0.01, **Figures 4A** **–E**). The same trend was observed for low doses of quercetin; however, the increase in stride frequency was not significantly (**Figure 4B**). These findings suggest that quercetin improves balance motion ability, subsequently preventing falls and reducing the fracture risk often experienced with osteoporosis.

### Quercetin Improves Morphological Changes in the Gastrocnemius Muscle Upon Orchiectomy

**Figure 5:**
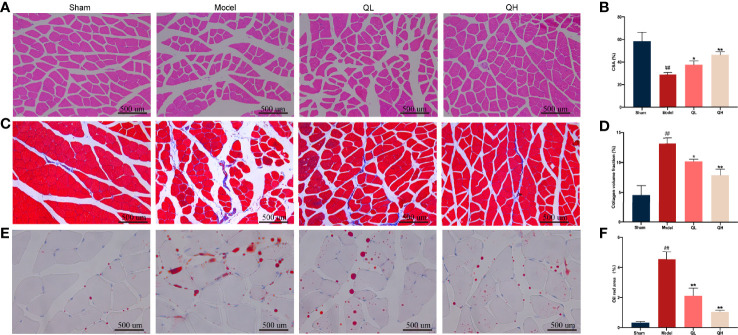
Effects of quercetin on morphological changes in the gastrocnemius muscle of mice that underwent orchiectomy. **(A)** Morphological changes in the gastrocnemius, visualized by H&E staining, and **(B)** changes in the average cross-sectional area of myofibers (CSA). **(C)** Masson’s staining of the gastrocnemius muscle, and **(D)** changes in the collagen fiber area. **(E)** Oil Red O staining of the gastrocnemius muscle, and **(F)** the changes in the area of the lipid droplet. ##*p<*0.01 vs. sham group; *p<0.05, **p<0.01 vs. model group.

To examine the effect of quercetin on morphological changes, H&E staining of the gastrocnemius muscle was performed (**Figures 5A, B**). The average cross-sectional area of myofibers decreased notably in the model group, along with an obvious broadening of the interstitial spaces among the muscle fibers. Furthermore, the cross-sectional area of the myofibers was significantly higher in the low- and high-quercetin groups than in the model group (*p*<0.05 and *p*<0.01, respectively), indicating that fiber atrophy was attenuated. We further used Masson’s staining to visualize the changes in the collagen fiber area. We found that the percentage of the collagen fiber area of gastrocnemius muscle in the model group was significantly higher than that in the sham-operated group (*p*<0.01), whereas there was an evident decrease in the collagen fiber area percentage of the low- and the high-quercetin groups (*p*<0.05 and *p*<0.01, respectively, **Figures 5C, D**). Finally, lipid accumulation in the gastrocnemius muscle was assessed by Oil Red O staining, and the results showed that the area of lipid droplets in the low- and high-quercetin groups was smaller than that in the model group (*p*<0.01, **Figures 5E, F**). These results indicate that quercetin may promote morphological changes in the gastrocnemius muscle by improving muscle mass and decreasing collagen fiber area and lipid droplets.

### Quercetin Alleviates Bone Loss and Increases Bone Formation After Orchiectomy in Mice

**Figure 6:**
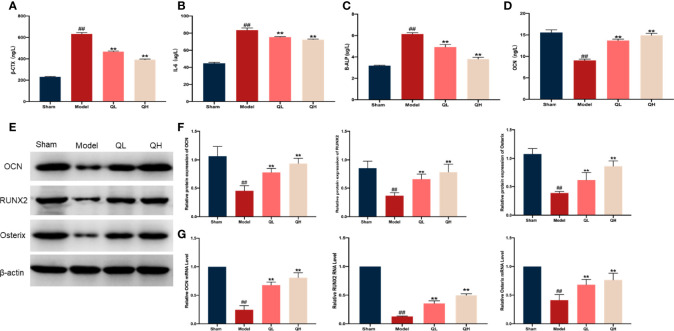
Effects of quercetin on bone metabolism in mice that underwent orchiectomy. The serum levels of markers of bone resorption including **(A)** β-isomerized C-terminal telopeptides (β-CTX), **(B)** interleukin 6 (IL-6), **(C)** bone alkaline phosphatase (B-ALP), and **(D)** osteocalcin (OCN). **(E)** The expression of bone formation proteins in the tibia, including OCN, RUNX2, and Osterix. **(F)** Semi-quantification of OCN, RUNX2, and Osterix by Image J software. **(G)** The mRNA expression of OCN, RUNX2, and Osterix in tibia. ##*p<*0.01 vs. sham group; **p<0.01 vs. model group.

To determine the impact of quercetin on bone homeostasis in mice that had undergone orchiectomy, markers of bone resorption (β-CTX, and IL-6) and bone formation (OCN and B-ALP) were evaluated using ELISA. Following orchiectomy, the serum levels of β-CTX, and IL-6 were markedly elevated in the model group when compared with the sham group (*p*<0.01). However, after 8 weeks of quercetin treatment, levels of these markers were decreased in the mice (*p*<0.01, **Figures 6A, B**). The serum levels of OCN significantly decreased whereas B-ALP increased after orchiectomy (**Figures 6C, D**). Quercetin treatment induced a significant increase in OCN and decrease in B-ALP (*p*<0.01). Moreover, the mRNA and protein expression of OCN, RUNX2, and Osterix were also analyzed (**Figures 6E** **–G**). The protein levels of OCN, RUNX2, and Osterix were significantly upregulated after 8 weeks of quercetin treatment (*p*<0.01, **Figures 6E, F**) as were the mRNA levels (*p*<0.01, **Figure 6G**).

### Quercetin Improves Lipid Metabolism Upon Orchiectomy

**Figure 7:**
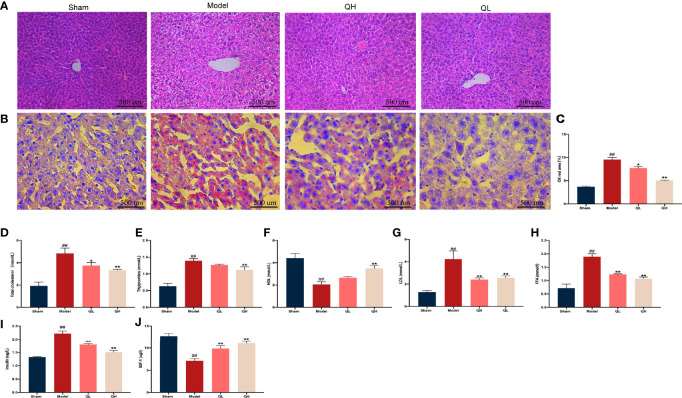
Effects of quercetin on lipid and glucose metabolism in mice that underwent orchiectomy. **(A)** The effects of quercetin on liver tissue morphology, visualized by H&E staining. **(B)** Oil-Red O staining of the liver, and **(C)** the percentage of lipid droplet area analysis. The serum levels of markers of lipid metabolism including **(D)** total cholesterol, **(E)** triglycerides, **(F)** high-density lipoprotein (HDL), **(G)** low-density lipoprotein (LDL), and **(H)** free fatty acid (FFA). The serum levels of markers of glucose metabolism including **(I)** insulin, and **(J)** insulin-like growth factor-1 (IGF-1). ##*p<*0.01 vs. sham group; *p<0.05, **p<0.01 vs. model group.

**Figure 8:**
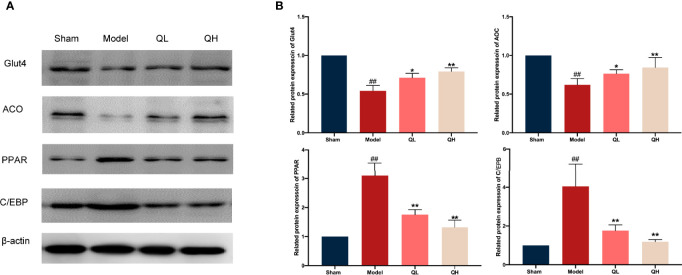
Changes in lipid and glucose metabolism in mice that underwent orchiectomy after quercetin treatment. **(A)** Expression of lipid and glucose metabolism proteins in the liver, including Glut4, ACO, PPAR, and C/EBP. **(B)** Glut4, ACO, PPAR, and C/EBP were semi-quantified by Image J software ##*p<*0.01 vs. sham group; *p<0.05, **p<0.01 vs. model group.

Histological abnormalities in liver upon orchiectomy and quercetin treatment were evaluated. As shown in **Figure 7A**, the structure of the liver was normal in the sham-operated group, whereas the fat vacuole, which is a typical sign of fat degeneration, was clearly observed in the model group. Furthermore, the area of lipid droplets in the model group was markedly enlarged compared to that in the sham-operated group. However, hepatic steatosis was significantly attenuated, and the area of lipid droplets was smaller after quercetin intervention (**Figures 7B, C**). After orchiectomy, the serum levels of triglycerides, total cholesterol, LDL, and FFA were significantly increased in the model group (*p*<0.01), whereas HDL levels were decreased (*p*<0.01). However, following quercetin treatments, total cholesterol, triglycerides, LDL, and FFA were significantly decreased, whereas HDL was increased in the low- and high-quercetin groups (**Figures 7D** **–H**). The mild change in triglycerides and HDL in the low-quercetin group was not statistically significant (**Figures 7E, F**). We further examined the effects of quercetin on key proteins related to lipid metabolism, by analyzing the expression of Cebp/β and Ppar-γ. As shown in **Figure 8**, the protein levels of Cebp/β and Ppar-γ significantly decreased after 8 weeks of quercetin treatment. These results indicated that quercetin effectively alleviated hepatic steatosis and improved lipid metabolism.

### Quercetin Improves Glucose Metabolism Upon Orchiectomy

In addition to lipid metabolism, we analyzed the corresponding glucose metabolism. After quercetin treatment, we observed a significant decrease in insulin and an increase in insulin-like growth factor-1 levels in the low- and high-quercetin groups (**Figures 7I, J**). Meanwhile, the expression of GLUT4 and ACO, which are major proteins related to glucose metabolism, as well as Cebp/β and Ppar-γ, key factors in lipid metabolism, was analyzed (**Figures 8A, B**). GLUT4 and ACO showed an increased expression upon quercetin treatment (*p*<0.05 for QL and *p*<0.01 for QH, **Figure 8B**). Protein levels of Cebp/β and Ppar-γ significantly decreased after 8 weeks of quercetin treatment (*p*<0.01). These results indicated that quercetin effectively alleviated hepatic steatosis and improved both glucose and lipid metabolism.

### Quercetin Modulates the GPRC6A/AMPK/mTOR Signaling Pathway

**Figure 9:**
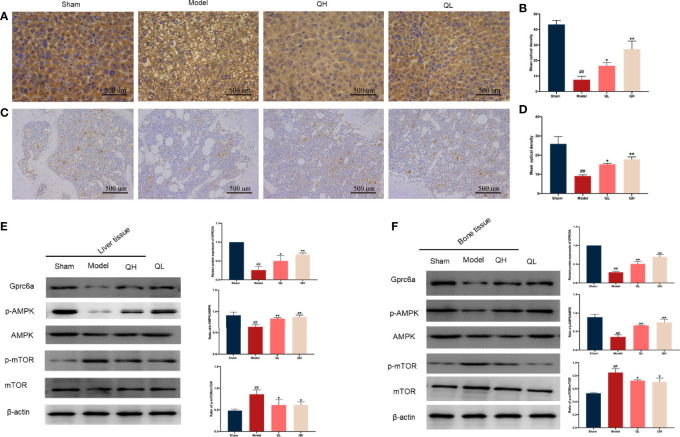
Effects of quercetin on the GPRC6A/AMPK/mTOR signaling pathway in mice that underwent orchiectomy. Immunohistochemical staining for GPCR6A and staining quantification in liver **(A, B)** and **(C, D)**. Expression of GPCR6A in the liver **(E)** and bone **(F)** evaluated by western blotting assay (on the left) and semi-quantitative analysis (on the right). ##*p<*0.01 vs. sham group; *p<0.05, **p<0.01 vs. model group.

As an essential modulated regulator of metabolism, GPRC6A may play a critical role in osteoporosis. To further elucidate the possible mechanisms by which this occurs, the expression of GPRC6A in the liver and femur was analyzed. As shown in **Figure 9**, GPRC6A was broadly expressed in the liver and femur tissues, and its expression in both tissues decreased significantly after orchiectomy (*p*<0.01, **Figures 9A-D**). Following quercetin treatment, GPRC6A expression increased again (*p*<0.05 for QL and *p*<0.01 for QH, for both tissue types). Next, the downstream signaling of GPRC6A, including AMPK and mTOR, was examined in both the liver (**Figure 9E**) and femur (**Figure 9F**). We found that the phospho-AMPK/AMPK ratio was elevated in the low- and high-quercetin groups, whereas the phospho-mTOR/mTOR ratio was reduced. These results indicated that quercetin may promote AMPK phosphorylation and inhibit mTOR phosphorylation. Furthermore, quercetin may exert biological effects on osteoporosis with metabolic disorders and may be associated with the GPRC6A/AMPK/mTOR signaling pathway.

## Discussion

Among other symptoms, testosterone deficiency, due to either chronic illnesses or endocrine treatments, can result in bone mineral density (BMD) decline. Despite its beneficial effects on bone health, testosterone replacement therapy may cause other problems, including decreased sperm production and fertility, cardiovascular disease, and prostate cancer or hyperplasia. In this study we aimed to determine the bone-protective effects of quercetin in mouse model of testosterone deficiency-induced osteoporosis. In contrast to previous investigations on rats, where osteoporosis manifests at 12 weeks after orchiectomy (22), we found that it appears at 8 weeks after orchiectomy in mice. Due to testosterone deficiency, the mice that underwent orchiectomy not only exhibited decreased bone density, bone mass, and imbalance of bone metabolism, but also endocrine hormone disorders. These results are consistent with clinical presentations and may provide the foundation for subsequent studies.

The potential pharmacological effects of quercetin have been extensively explored as dietary constituents. Due to its antioxidative, anti-inflammatory, and anti-apoptotic effects, it has been clinically applied for the treatment of various diseases (11–15). In this study, we found that quercetin increased bone mass and improved bone strength in orchiectomy mice, indicating that quercetin has therapeutic effects on osteoporosis induced by testosterone deficiency. Furthermore, the effect of quercetin in musculoskeletal system has been described here for the first time. In this study, quercetin was confirmed to significantly promote histological changes by improving the cross-sectional area of the myofibers, decreasing collagen fiber area and lipid droplets in gastrocnemius muscle. In parallel, gait analysis results showed that quercetin could improve balance motion ability. These findings suggest that quercetin may have potential of reducing the fracture risk often experienced with osteoporosis.

Conventionally, testosterone affects bone mass and strength *via* its conversion to 5α-dihydrotestosterone in peripheral tissues. Then, 5α-dihydrotestosterone further promotes osteoblast proliferation and inhibits osteoblast apoptosis by interacting with the androgen receptor. When the androgen receptor signaling is blocked, the mice exhibits a series of abnormal musculoskeletal findings, including decreased bone volume and trabecular number (23). Previous studies have found that orchiectomy is often accompanied by decreased levels of testosterone and increased bone loss (24). Similarly, we found that markers for bone resorption, TRAP, β-CTX, and IL-1, were significantly elevated after orchiectomy. In addition, the correlated decrease in markers for bone resorption and the ratio of OCs/BS and N.OC/BS indicated that quercetin treatments alleviated trabecular bone loss in the distal femur after orchiectomy.

B-ALP is a ubiquitous metalloenzyme that catalyzes the hydrolysis of phosphate esters and generates an organic radical and inorganic phosphate. As a mature and active marker of osteoblasts, B-ALP is involved in bone formation and reflects osteocyte formation and activity status (25). As expected, the serum B-ALP levels increased gradually after orchiectomy. This suggests that testosterone deficiency impaired osteocyte function, and bone metabolism was in a high bone turnover state. The biochemical marker of bone turnover, OCN, principally reflects the bone formation produced by mature osteoblasts. In addition to being essential for mineralized bone matrix, OCN also stimulates testosterone biosynthesis *via* uOCN (26). Several researchers have found that OCN^–/–^ mice exhibit severe hypergonadotropic hypogonadism, including small testes and impaired fertility (27). Additionally, a series of studies reported a close relationship among testosterone and OCN in patients with metabolic disorders (28–30). We found in this study that the levels of OCN and uOCN in serum decreased significantly following orchiectomy, which implies that the relationship between testosterone and OCN is not one-way but includes a network or coupling system. In the testes, 95% of testosterone is produced, with the remaining 5% secreted by the adrenal glands. Recent research has shown that the main mechanism by which quercetin increases testosterone is by promoting its synthesis in the Leydig cells (15). Notably, quercetin significantly increased the serum levels of testosterone, OCN, and uOCN after orchiectomy. On the one hand, the increase in testosterone suggests that quercetin does increase testosterone by promoting synthesis in the adrenal glands, whereas the increase in uOCN reflects, at least in part, that quercetin may also increase testosterone by promoting OCN synthesis.

As a cornerstone of treatment methods, orchiectomy has been widely applied and may be the most crucial cause of accelerated bone loss in prostate cancer (31). Previous studies have suggested that the level of serum testosterone is correlated with long-term survival rate in prostate cancer (32). Nevertheless, the decreasing serum levels of testosterone with orchiectomy were consistent with its clinical presentation after ADT, and quercetin increased testosterone levels in the serum. This may suggest that elevated serum testosterone levels might increase the possibility of recurrence in patients with prostate cancer. The importance of measuring changes in testosterone levels in patients undergoing ADT is undoubtedly important. However, a recent systematic review on the relationship between testosterone and prostate cancer reported that low serum levels of testosterone might enhance the risk of prostate cancer (33). These contradictions might have multiple explanations, including the fact that serum testosterone is not equivalent to intraprostatic testosterone, the indeterminate effects of androgen receptors, and the different testosterone measurement sites between serum and intraprostatic region (34). Furthermore, the contraindications for testosterone replacement in prostate cancer have been challenged. Several studies have suggested that the risk of exogenous testosterone replacement appears to be small and may reduce biochemical recurrence in prostate cancer following a prostatectomy (35–38). In addition, various studies have directly confirmed that intervention with 75–150 mg/kg of quercetin may inhibit tumor growth in mice with prostate cancer (39–42). This finding indicates that there is no additional risk of prostate cancer after quercetin treatment. Overall, based on the antitumor efficacy, quercetin could improve bone characteristics in osteoporosis due to testosterone deficiency.

As a main anabolic steroid hormone, testosterone exerts biological effects by regulating the energy balance to maintain glucose and lipid synthesis (43). Previous studies have shown that testosterone deficiency significantly alters glucose and lipid metabolism. In a prospective study of 177 patients who received ADT treatment for one year, total testosterone level decreased by 97.0%, but total cholesterol, LDL, and triglyceride levels increased by 10.6, 14.3, and 16.2%, respectively (44). In addition to alterations in triglyceride, cholesterol, LDL, and HDL levels, a decrease in FFA levels was also observed in our study. Consistently with previously reported data (45, 46), the elevation of circulating insulin level and lower serum level of IGF-1 indicates that orchiectomy induced insulin resistance. Testosterone deficiency often accounts for negative effects on lipid and glucose metabolism, and some bone and skeletal metabolism comorbidities may be associated with these changes. Abnormal bone metabolism can cause disturbed glucose metabolism by reducing the advanced glycation end product (AGE) (47). Aung and Lee found that inhibiting AGE and regulating glucose homeostasis may promote osteogenic differentiation of bone marrow mesenchymal stem cells (BMSCs) and improve bone metabolism and mass (47, 48). A recent study also found that orchiectomy could lead to lipid metabolism disorder, and decreased bone characteristics were improved after testosterone supplementation to decrease blood lipid levels. These findings show that glucose and lipid metabolic disturbances are risk factors for osteoporosis due to testosterone deficiency.

Consistently with computer simulations (19), except for increasing protein levels of molecules involved in glucose uptake (GLUT4) and decreasing proteins involved in lipid production (PPAR, C/EBP), quercetin significantly promoted the expression level of GPRC6A in the liver. GPRC6A is activated by OCN and testosterone and performs an essential function in the modulation of testosterone and energy metabolism (18). Due to lack of OCN and testosterone, decreased expression of GPRC6A was simultaneously detected in orchiectomy mice. After knockout of GPRC6A in the hepatocytes, male mice exhibited several metabolic abnormalities, including abnormal glucose levels and increased serum FFA and cholesterol levels (49). Many of our findings are consistent with this study, which may indicate that GPRC6A is a potential therapeutic target for metabolic disorders in orchiectomy mice. To date, the downstream targets of quercetin-GPRC6A have not yet been identified. However, with intervention of quercetin, we found that the phospho-mTOR/mTOR ratio decreased gradually as the phospho-AMPK/AMPK ratio increased. The serine/threonine kinase, AMPK, that is phosphorylated in response to a decrease in AMP/ATP ratio, is an essential regulatory factor in glucose and lipid metabolism. Glut 1 and Glut 4 are the major insulin-sensitive glucose transporters, and they have been demonstrated to be stimulated by AMPK and promote glucose transfer into the cell (50). In addition, phosphorylated AMPK alleviates endoplasmic reticulum stress and increases hepatic autophagy to reduce the levels of hepatic cholesterol and triglycerides by inhibiting the phosphorylation levels of mTOR (51). These results indicate that quercetin regulates glucose metabolism and inhibits lipid metabolism *via* the GPRC6A/AMPK/mTOR signaling pathway.

Furthermore, PPAR and C/EBP were obviously downregulated, whereas RUNX2 and Osterix were markedly upregulated after quercetin treatment. Phosphorylated AMPK could significantly increase osteogenic differentiation by promoting RUNX2 expression and inhibiting PPAR expression, which is an indicator of differentiation from BMSCs to osteoblasts and adipocytes, respectively (52, 53). These findings initiated our further analysis of whether quercetin could regulate BMSC differentiation by regulating the GPRC6A/AMPK/mTOR signaling pathway. We found that GPRC6A was expressed in the bone tissues, and its expression decreased after orchiectomy. Furthermore, similar to its expression in the liver, changes in p-AMPK/AMPK and p-mTOR/mTOR ratios suggested that quercetin could inhibit adipogenic differentiation and promote osteogenic differentiation by directly regulating the GPRC6A/AMPK/mTOR signaling pathway. We found that GPRC6A was expressed in the bone tissues, and its expression decreased after orchiectomy. Furthermore, similar to its expression in the liver, changes in phospho-AMPK/AMPK and phospho-mTOR/mTOR ratios suggested that quercetin could inhibit adipogenic differentiation and promote osteogenic differentiation by directly regulating the GPRC6A/AMPK/mTOR signaling pathway.

## Conclusion

In summary, we first verified the biological function of quercetin in testosterone deficiency-induced osteoporosis. We showed that 75 mg/kg/d and 150 mg/kg/d of quercetin partially improved bone quality through modulation of bone metabolism and bone microarchitecture in mice that had undergone orchiectomy. The beneficial effects of quercetin might be related to its ability to regulate glucose and lipid metabolism *via* the GPCR6A/AMPK/mTOR signaling pathway. These findings may act as a foundation for the further examination of quercetin as great potential drug for osteoporosis induced by testosterone deficiency.

## Data Availability Statement

The raw data supporting the conclusions of this article will be made available by the authors, without undue reservation. (NO. ACU170804).

## Ethics Statement

The animal study was reviewed and approved by Experimental Animal Center of Nanjing University of Chinese Medicine.

## Author Contributions

JS and WD conceived and designed the experiment, analyzed the data and interpreted the results and developed the manuscript. YP, PT, CW, XL, and QH collaborated in the pharmacological experiments. YM and YG supervised the work and proofread the manuscript. All authors contributed to the article and approved the submitted version.

## Funding

This work was supported by National Natural Science Foundation of China (No.81904229, 82074458, 81973881), Traditional Chinese and Western Medicine Clinical Medicine Brand Construction Project of Jiangsu Higher Education Institutions (Phase II)(2020PPZXL261), 2021 Future plans of Shanghai Municipal Hospital of Traditional Chinese Medicine(No.WLJH2021ZY-ZLZX001/GZS001/MZY034) and was also supported by the Postgraduate Research and Practice Innovation Program of Jiangsu Province (KYCX21_1646).

## Conflict of Interest

The authors declare that the research was conducted in the absence of any commercial or financial relationships that could be construed as a potential conflict of interest.

## Publisher’s Note

All claims expressed in this article are solely those of the authors and do not necessarily represent those of their affiliated organizations, or those of the publisher, the editors and the reviewers. Any product that may be evaluated in this article, or claim that may be made by its manufacturer, is not guaranteed or endorsed by the publisher.
